# Dysphagia from an aberrant internal carotid artery: a case report

**DOI:** 10.1007/s00405-025-09417-6

**Published:** 2025-05-07

**Authors:** Marie Bjerg Larsen, Pádraig O’Leary

**Affiliations:** Department of Oto-Rhino-Laryngology, Head and Neck Surgery, Aarhus University Hospital, 8200 Aarhus, Denmark

**Keywords:** Dysphagia, Internal carotid artery, Occupational therapy

## Abstract

**Purpose:**

We present a case of dysphagia caused by an aberrant internal carotid artery (ICA). By reporting this rare occurrence, we hope to highlight the anomaly as a differential in cases of persistent, progressive dysphagia.

**Results:**

Even though the symptomatic mass effect of the ICA warranted the option of surgical intervention, due to a patient-centered approach with an emphasis on personal preference, the patient was instead referred for specialized ergotherapy.

**Conclusion:**

This case characterizes a rare yet significant cause of dysphagia, in addition, it illustrates the necessity of a multidisciplinary approach when dealing with complex cases of dysphagia.

## Introduction

We report a case of dysphagia caused by an aberrant internal carotid artery (ICA). Although anatomical variations in the ICA have a reported incidence of approximately 10–40% [1], displacement severe enough to cause hypopharyngeal compression and dysphagia is rare. This report aims to highlight this anomaly as a differential diagnosis in patients with persistent, progressive dysphagia. Accurate diagnosis is essential because surgical intervention is commonly offered for anatomical causes of dysphagia. However, when dysphagia arises due to an aberrant ICA, the high risk of catastrophic bleeding associated with surgery must be carefully considered in treatment planning.

## Case

An 76 year old woman was referred to the Clinic for Swallowing Disorders at Aarhus University Hospital by her general practitioner. The patient had comorbidities including goiter, hypertension, a history of smoking, mildly impaired renal function, and significant aortic stenosis. She underwent transcatheter aortic valve implantation in April 2021 with a successful outcome, maintaining good biventricular function and valve performance post-procedure. At the time she was presenting with progressive dysphagia over several years, initially presumed to have Zenker’s Diverticulum. She reported daily incidents of food becoming lodged in her throat and several occurrences of transient airway obstruction. Socially, her dysphagia was impactful, as she avoided eating in public due to frequent regurgitation, particularly when lying down. She did not report weight loss.

**Fig. 1:**
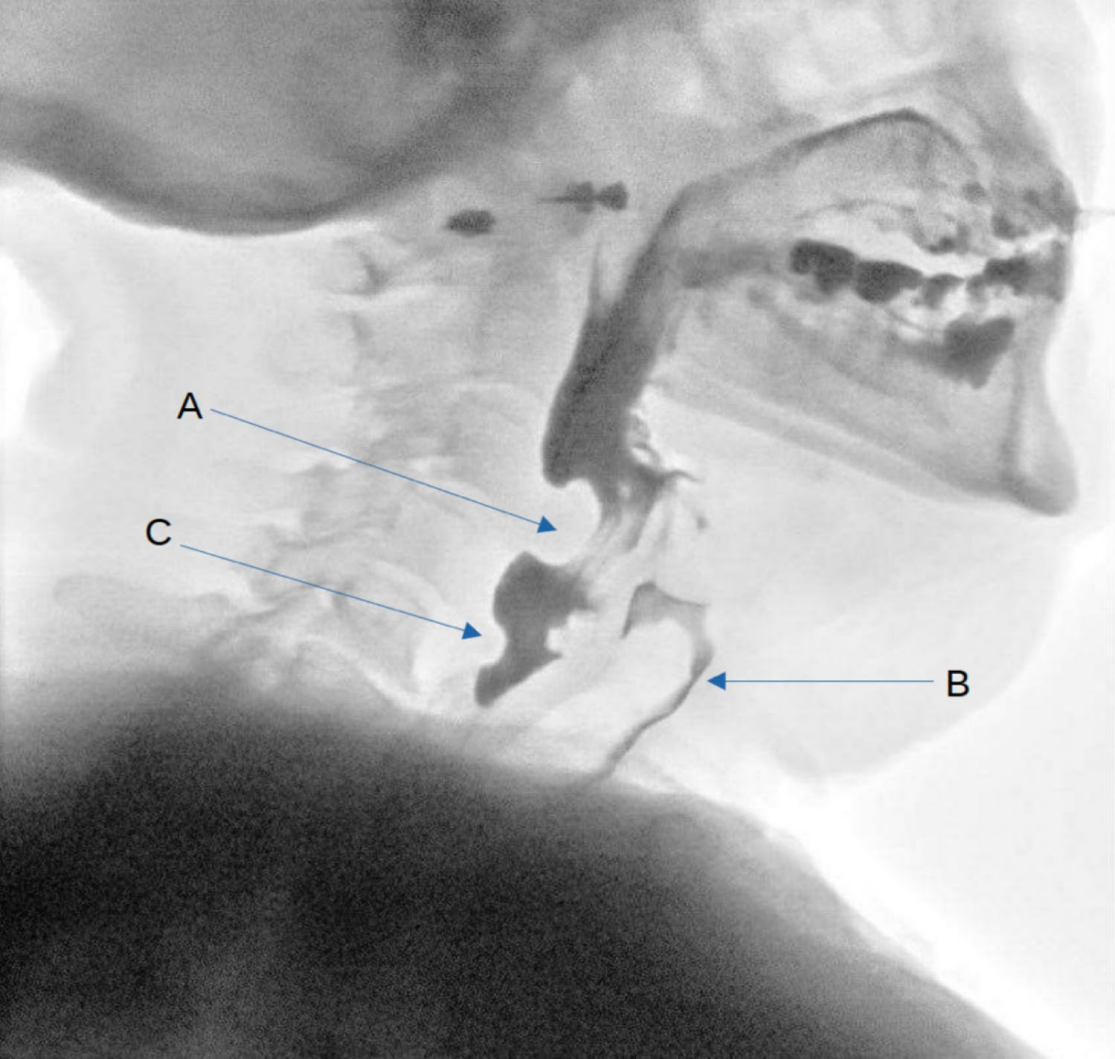
A screenshot of the MBS examination, sagittal view. **A** Impression of the right-sided ICA, **B** Bolus aspiration to the airway, **C** Minor cricopharyngeal bar

**Fig. 2:**
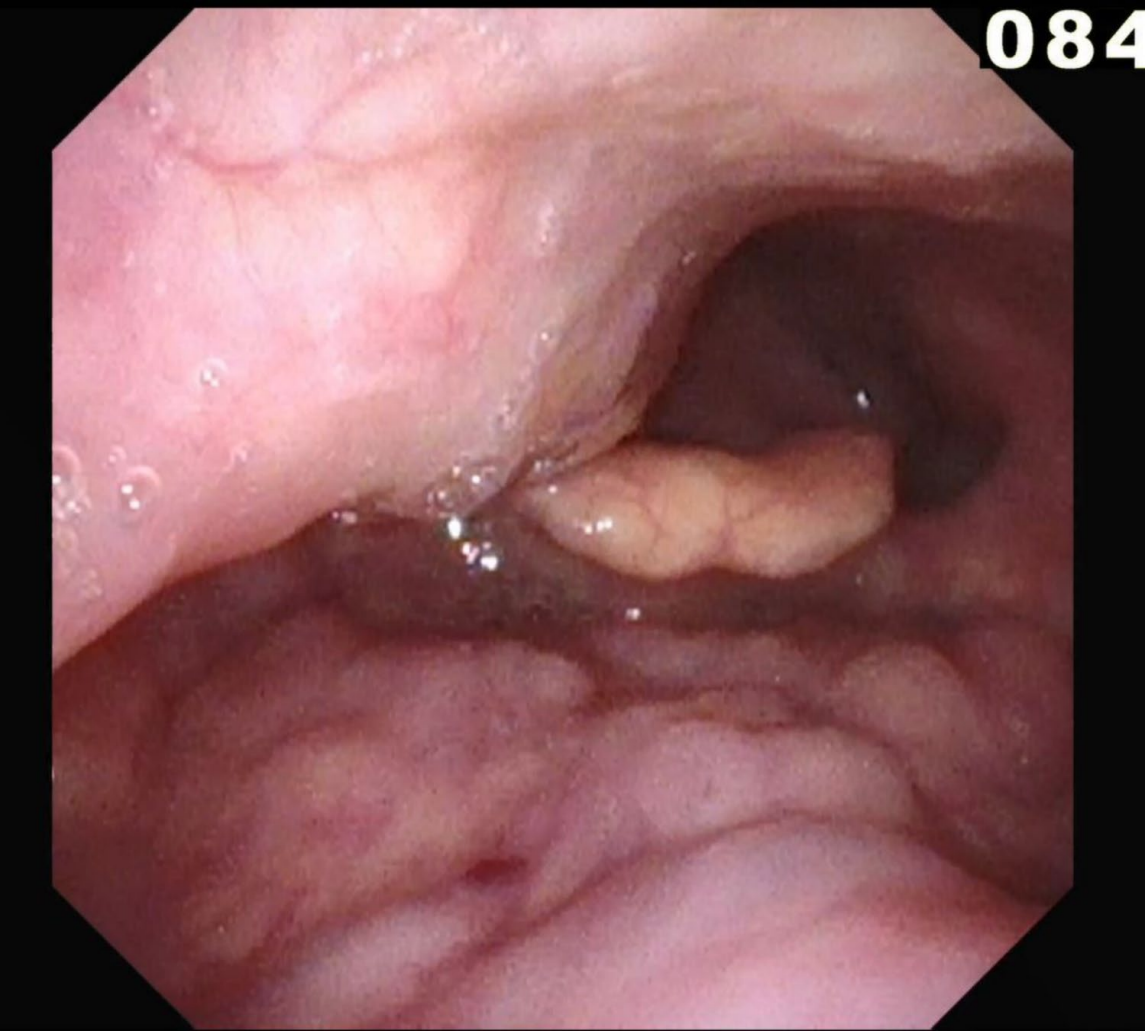
A screenshot from the FEES test showing retention in the vallecula

Upon fiberoptic evaluation, a large pulsatile structure was visible on the right side of the oropharynx, occupying significant space in both the oropharynx and hypopharynx. A video fluoroscopic swallowing exam with barium contrast (Modified Barium Swallowing test (MBS)) and apple purée revealed a cricopharyngeal bar (CP-bar) at the upper esophagus, causing bolus retention (Fig. 1). The test with apple purée showed dyscoordination in oral processing and premature bolus leakage, while barium testing led to immediate aspiration, requiring termination of the exam. A Flexible Endoscopic Evaluation of Swallowing (FEES) further revealed highly dyscoordinated swallowing with premature bolus entry into the pharynx and residual retention in the piriform sinus and vallecula (Fig. 2).

**Fig. 3:**
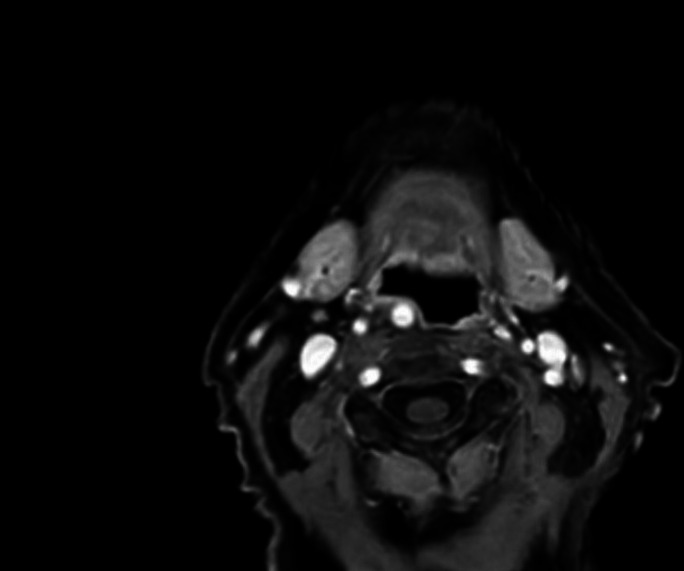
MRI, axial view. Impression of the right-sided ICA displacing the hypopharynx

**Fig. 4:**
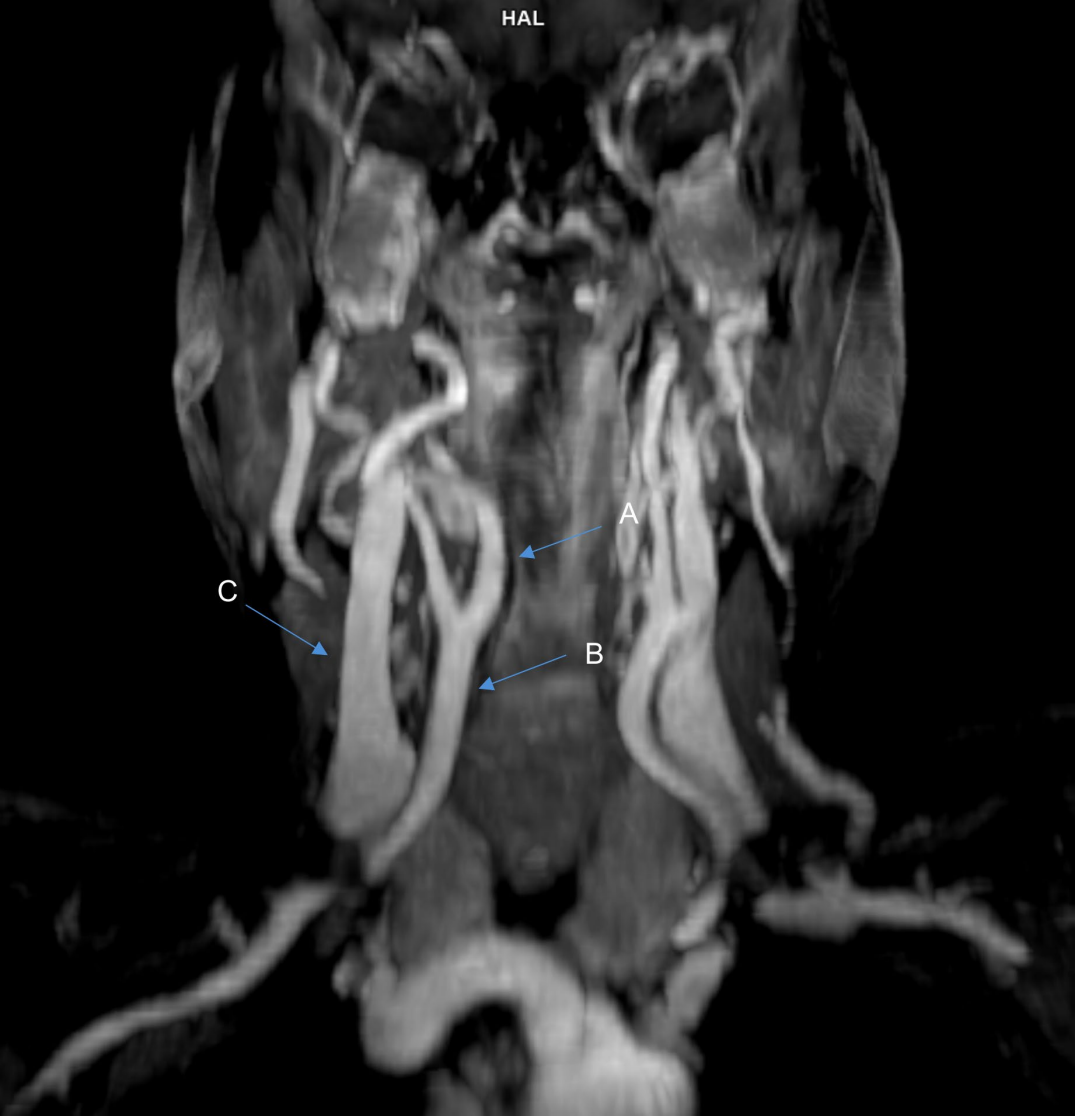
MRI, coronal view. The course of the common carotid artery and the ICA in the neck, **A** ICA, right-sided, **B** Commom carotid artery, right-sided, **C** Internal jugular vein, right-sided

The patient was subsequently referred for MRI, which confirmed an aberrant right-sided ICA displacing the hypopharynx (Figs. 3 and 4). Given the high bleeding risk associated with an aberrant ICA, surgical intervention was considered too dangerous. The patient decided against esophageal dilation or myotomy of the cricopharyngeal bar after discussing the potential risks with the ENT specialist. Instead, she was referred to a specialized deglutition therapy program to address her symptoms.

## Discussion/Conclusion

Many procedures in the ENT setting carry a potential risk of injury to the ICA, including tonsillectomies and similar procedures. Knowledge of the course of the ICA and common anatomical variations has therefore been of interest for a long time. As early as 1868, Henle described variations in the course of the ICA [2]. The ICA develops from the third branchial arch artery and is initially coiled until the descent of the large vessels and the heart into the mediastinal space during further embryological development. Under normal circumstances, this process elongates and straightens the ICA [2]. Variations during this phase may result in a congenitally aberrant ICA with an anteromedial curvature at the level of the oropharynx [3]. Other anatomical anomalies have been attributed to degenerative changes; Paulsen et al. found an increased prevalence of both kinking and curving of the vessel in older age [3].

Aberrant internal carotid arteries are uncommon causes of dysphagia [1, 4–6]. Although a parapharyngeal course of the extracranial ICA occurs in 10–40% of cases, it rarely results in significant, symptomatic displacement [1]. Besides dysphagia, symptoms of an aberrant ICA can include dysphonia, cough, and foreign body sensation [5]. In certain instances, alterations in blood flow due to abnormal ICA positioning may lead to thromboembolic events, causing neurological symptoms [4].

In cases with symptomatic mass effects, whether to pursue surgical intervention depends on the patient’s symptoms, surgical risks, and personal preferences. Some cases report vascular surgical solutions for severe quality-of-life impacts, while others, including our case, demonstrate the value of conservative management [4, 6]. with reference to their risk of bleeding [3]. In this case, vascular surgery was not recommended. After counselling with the ENT specialist, the patient opted against oesophageal dilatation or myotomy of the cricopharyngeal bar. Instead, involvement of deglutition therapists (occupational therapists) was deemed a more favourable approach for managing the patient’s condition.

This case highlights the importance of a multidisciplinary approach involving ENT, radiology, and deglutition therapeutic intervention to manage complex dysphagia. Here, the patient-centered decision to avoid surgical risks reflects a comprehensive, individualized treatment plan emphasizing safety and quality of life.

## Data Availability

Data available on request due to privacy/ethical restrictions.The data that support the findings of this study are available on request from the corresponding author, M. B. Larsen.The data are not publicly available due to restrictions e.g. their containing information that could compromise the privacy of research participants.
